# Gray Matter Involvement in Radiologically Isolated Syndrome

**DOI:** 10.1097/MD.0000000000003208

**Published:** 2016-04-01

**Authors:** Andrés Labiano-Fontcuberta, Virginia Mato-Abad, Juan Álvarez-Linera, Juan Antonio Hernández-Tamames, Mª Luisa Martínez-Ginés, Yolanda Aladro, Lucía Ayuso, Ángela Domingo-Santos, Julián Benito-León

**Affiliations:** From the Department of Neurology, University Hospital “12 de Octubre” (AL-F, AD-S, JB-L); Neuroimaging Laboratory, Center for Biomedical Technology, Rey Juan Carlos University, Móstoles (VM-A, JAH-T); Department of Radiology, Hospital Ruber International (JA-L); Department of Neurology, University Hospital “Gregorio Marañón,” Madrid, Spain (MLM-G); Department of Neurology, University Hospital of Getafe, Getafe (YA); Department of Neurology, University Hospital “Principe de Asturias,” Alcalá de Henares (LA); Centro de Investigación Biomédica en Red sobre Enfermedades Neurodegenerativas (CIBERNED) (JB-L); and Department of Medicine, Complutense University (JB-L), Madrid, Spain.

## Abstract

The unanticipated magnetic resonance imaging (MRI) detection in the brain of asymptomatic subjects of white matter lesions suggestive of multiple sclerosis has recently been named as radiologically isolated syndrome (RIS). The pathophysiological processes of RIS remain largely unknown and questions as to whether gray matter alterations actually occur in this entity are yet to be investigated in more detail. By means of a 3 T multimodal MRI approach, we searched for cortical and deep gray matter changes in a cohort of RIS patients. Seventeen RIS patients, 17 clinically isolated syndrome (CIS) patients (median disease duration from symptom onset = 12 months), and 17 healthy controls underwent MRI and neuropsychological testing. Normalized deep gray matter volumes and regional cortical thickness were assessed using FreeSurfer. SIENAX was used to obtain normalized global and cortical brain volumes. Voxelwise morphometry analysis was performed by using SPM8 software to localize regions of brain tissue showing significant changes of fractional anisotropy or mean diffusivity. Although no differences were observed between CIS and healthy controls groups, RIS patients showed significantly lower normalized cortical volume (673 ± 27.07 vs 641 ± 35.88 [cm^3^ × 10^3^, Tukey *P* test = 0.009) and mean thalamic volume (0.0051 ± 0.4 vs 0.0046 ± 0.4 mm, *P* = 0.014) compared with healthy controls. RIS patients also showed significant thinning in a number of cortical areas, that were primarily distributed in frontal and temporal lobes (*P* < 0.05, uncorrected). Strong correlations were observed between T2-white matter lesion volume and regional cortical thickness (rho spearman ranging from 0.60 to 0.80). Our data suggest that white matter lesions on T2-weighted images are not the only hallmark of RIS. Future longitudinal studies with larger samples are warranted to better clarify the effect of RIS-related white matter lesions on gray matter tissue.

## INTRODUCTION

The steady increase in the use of magnetic resonance imaging (MRI) for the evaluation of medical conditions, such as headaches or dizziness, has led to the emergence of a new condition named radiologically isolated syndrome (RIS), which is characterized by incidental brain MRI finding of white matter lesions demonstrating dissemination in space in subjects with a normal neurologic examination, and without historical accounts of typical multiple sclerosis (MS) symptoms.^1^

A number of recent studies suggest that RIS and MS patients share both nonmotor clinical features^2–5^ and quantitative brain tissue damage,^6–10^ thereby suggesting that RIS, as an entity, reflects the earliest and preclinical form of MS. In line with this assumption, approximately one-third of RIS patients, mainly those with spinal cord involvement, are at higher risk for future symptomatic demyelinating event within 5 years.^11^

There are too many unanswered questions regarding this entity, with the current impossibility of clarifying whether the observed white matter changes reflect the earliest stage of MS, a nondisabling form of MS, or even a different pathological condition. Furthermore, RIS quantitative MRI studies to date have either included a meaningful proportion of RIS patients with markers of evolution to future MS,^6,8,12^ and as a consequence, their results might not be entirely extrapolable to the general RIS population, or have compared RIS patients with MS patients^3,8,12^ rather than with CIS patients who potentially might have a closer biologic and pathological similarity with RIS patients.

The great uncertainty over the true morphological changes in brain with RIS underlines the necessity of additional comprehensive studies with multimodal structural brain imaging analyses. Until now, no study has been conducted to compare the brain damage occurring beyond T2 visible lesions between RIS and CIS patients. The comparison of a group of RIS patients with a CIS group without high disease activity who have not converted to definite MS has the potential advantage to directly contrast 2 groups with a closer stage disease.

In this context, the present study used a multimodal 3 T MRI approach aimed to evaluate the differences in cortical thickness, cortical and subcortical gray matter volume, and white matter integrity between RIS, CIS, and healthy volunteers; asses to what extent RIS quantitative brain measures share similarities or differences with those of CIS patients; analyze the correlation between specific brain changes with cognitive and psychiatric symptoms of RIS patients.

## METHODS

### Study Design and Subjects

Seventeen RIS subjects, most of whom came from already existing MS databases, were recruited at 4 centers specialized in demyelinating diseases in Madrid (Spain). These subjects had come to our attention after undergoing conventional brain 1.5 T MRI for various medical events not suggestive of MS. All 1.5 T MRI examinations included axial fluid-attenuated inversion recovery, fast spin echo, and T2- and T1-weighted without and with gadolinium in axial, coronal, and sagittal planes of view. Brain white matter abnormalities were initially identified by a neuroradiologist and subsequently examined by an MS specialist at each clinical site to guarantee they fulfilled the Okuda MRI criteria for RIS,^1^ which imply the presence of white matter abnormalities suggestive of a demyelinating process (ovoid, well-circumscribed, and measuring >3 mm^2^) that satisfied Barkhof criteria (at least 3 of 4 criteria) for dissemination in space^13,14^; not better accounted for by other disease processes, such as, in particular, vascular disease; and no apparent impact on everyday functioning.

After the initial MRI imaging, all RIS patients underwent cervical spinal MRI, but only a subgroup (50%) agreed to undergo lumbar puncture for CSF analysis. An extensive neurologic examination and an accurate clinical history were performed by neurologists with expertise in MS (AL-F, MLM-G, YA, LA, AD-S, and JB-L) to rule out both any neurologic sign and history of remitting clinical symptoms lasting >24 h consistent with MS. In addition, they underwent a complete nonstandardized workup (sedimentation rate, anticardiolipin antibody screen, antineutrophil cytoplasmic antibodies, antinuclear antibodies, Borrelia and syphilis serologies, rheumatoid factor, serum angiotensin-converting enzyme, thyroid function studies, and vitamin B12 level) to rule out other medical conditions that could explain the observed lesions on brain MRI.

Seventeen patients who had presented with a CIS suggestive of MS were recruited from the University Hospital “Gregorio Marañón,” and from the University Hospital of Getafe, both in Madrid (Spain). Although the frequency of follow-up MRI examinations in CIS patients was slightly different across participating centers, all CIS patients underwent 1.5 T MRI examination (axial fluid-attenuated inversion recovery, fast spin echo, and T2- and T1-weighted without and with gadolinium in axial, coronal and sagittal planes of view) and a complete neurológic evaluation, including Expanded Disability Status Scale (EDSS)^15^ by experienced neurologists (MLM-G and YA). Dissemination in space and dissemination in time were evaluated according to the McDonald 2010 criteria.^16^ Patients selected met the following inclusion criteria: single clinical episode indicative of MS; total follow-up time of at least 3 months from the occurrence of the first inflammatory demyelinating event; and the presence of ≥1 asymptomatic T2 lesion(s) in at least ≥2 brain locations considered characteristic for MS (juxtacortical, periventricular, infratentorial, and spinal cord)^17^ at the initial or follow-up MRI. Participants were excluded if they had received steroid medication during the month before the study inclusion and a longitudinal evaluation >5 years.

We excluded RIS or CIS patients with history of alcohol or drug abuse, major acute comorbidities, or any major serious chronic illness 1 year before inclusion (patients with a stable chronic medical conditions were included).

RIS or CIS patients were matched for age, sex, and education with a control group consisting of 17 healthy controls (14 women, 3 men; mean age 42 years, range 27–56 years) with no history of known psychiatric or neurological disorders, which was recruited either from relatives or friends from health professionals at the University Hospital “12 de Octubre” of Madrid (Spain).

Once the study was described to subjects and written (signed) informed consent was obtained from all enrollees, a multisequence MRI examination was acquired in a single session using a single 3 T scanner at CIEN (Center for Research on Neurological diseases, in Spanish) Foundation in Madrid (Spain). Psychiatric and neuropsychological tests were conducted in a single session by experienced clinical neuropsychologists (VP; YH, MC, see acknowledgements) who were blinded to the clinical status during an interview during the week in which they had completed the aforementioned MR examination.

All procedures were approved by the ethical standards committees on human experimentation at the University Hospital “12 de Octubre” (Madrid).

### Measurement Instruments

Cognitive functioning was performed through the Rao Brief Repeatable Battery.^18^ The Stroop test was administered to evaluate executive functions.^19^ Participants also completed the Vocabulary and Matrix subtests from the Wechsler Adult Intelligence Scale–Third Edition (WAIS-III).^20^ Vocabulary subtest was used as a measure of crystallized intelligence, which is influenced by educational experience. Vocabulary scores have been suggested as a more reliable measure of cognitive reserve than demographic information (education and occupation),^21^ as some patients may have actually greater cognitive reserve through lifelong habits (ie, reading, writing, and high-demand activities) and often surpass patients who may have more years of formal education. Matrix subtest was used to measure fluid intelligence as it requires the ability to solve novel problems with minimal dependence on previously acquired knowledge.

Depression severity was measured by the original 17-item version of the Hamilton Depression Rating Scale.^22^

### MRI Acquisition

All MRI data were acquired with a clinical 3 T Signa HDx MRI scanner (GE Healthcare, Waukesha, WI) using an 8-channel phased array coil. The imaging (MRI) standardized protocol (without injection of contrast agent) included a 3D T1-weighted SPGR with a TR = 10.012 ms, TE = 4.552 ms, TI = 600 ms, NEX = 1, acquisition matrix = 288 × 288, full brain coverage, resolution = 0.4688 × 0.4688 × 1 mm, flip angle = 12.

The diffusion-weighted image (DWI) protocol acquisition consisted of 3 images without diffusion gradients (b = 0 s/mm^2^) followed by 45 images measured with 45 directions (b = 1000 s/mm^2^) isotropically distributed in space. Additional parameters of the acquisition were: TE = 85.3 ms, TR = 10.100 ms, flip-angle = 90, slice thickness = 3 mm (no gap), resolution = 2.6042 × 2.6042 × 2.6 mm, FOV = 250 mm and axial acquisition.

### Data Post-processing

#### MRI

Cortical thickness and cortical volume measurements were calculated using the freely available software FreeSurfer (http://surfer.nmr.mgh.harvard.edu/). Using a surface-based approach, FreeSurfer can automatically segment the brain into different cortical regions of interest, and calculate average thickness in the defined regions. In brief, images underwent preprocessing including intensity normalization and skull stripping, which was followed by labeling of cortical and subcortical regions. FreeSurfer's main cortical reconstruction pipeline begins with registration of the structural volume with the Talairach atlas.^23^ After bias field estimations and the removal of bias, the skull is stripped and subcortical white and gray matter structures were segmented.^24^ Next, tessellation, automated topology correction, and surface deformation routines create white/gray (white) and gray/cerebrospinal fluid (pial) surface models.^25^ These surface models were then inflated, registered to a spherical atlas, and used to parcellate the cortical mantle according to gyral and sulcal curvature.^26^ The closest distance from the white surface to the pial surface at each surface's vertex was defined as the thickness.^26^ Average cortical thickness, surface area, and total volume statistics corresponding to each parcellated region could then be computed. The accuracy of FreeSurfer's results was then assessed visually for the different subjects.

#### DWI

DWI data were preprocessed with FMRIB's Diffusion Toolbox (http://fsl.fmrib.ox.ac.uk/fsl/fslwiki/FslOverview/). Preprocessing consisted of eddy-current correction, motion correction, and the removal of non-brain tissue using the robust Brian Extraction Tool.^27^ Diffusion tensor images (DTIs) were created using the weighed least squares fitting method. We derived images of fractional anisotropy or mean diffusivity from the DTI. To calculate the specific fractional anisotropy or mean diffusivity values in the subcortical regions, we need to map the DTI space to the Freesurfer's structural space (described above). The fractional anisotropy or mean diffusivity maps were resampled by means of a rigid-body transformation from the diffusion to the structural space. After that, the fractional anisotropy or mean diffusivity mean and standard deviation values from the Freesurfer's subcortical regions were computed.

In addition, a voxel-based analysis pipeline was used to find differences in fractional anisotropy or mean diffusivity between groups. Voxel-based analysis of fractional anisotropy or mean diffusivity images were carried out with SPM8 software (www.fil.ion.ucl.ac.uk/spm/software/spm8/). First, b0 images were manually aligned to the anterior commissure-posterior commissure line, and the same alignment was applied to the fractional anisotropy or mean diffusivity images. Then, these fractional anisotropy images were coregistered to a fractional anisotropy template from FMRIB's Software Library using linear affine registration with normalized mutual information as the fitness function.^28^ The same spatial transformation was applied to the mean diffusivity maps. The registered images were normalized to the fractional anisotropy template using a nonlinear registration algorithm^29^ and were then smoothed with an 3D Gaussian kernel (4-mm full-wide half maximum). The spatial transformation was again applied to the mean diffusivity maps.

#### White Matter Lesion Volume Analysis

Study images were assessed by an experienced neuroradiologist (JA-L) who was unaware of participant clinical status. T2 hyperintense lesions were segmented in fluid-attenuated inversion recovery images by employing the automated lesion growth algorithm^30^ as implemented in the LST toolbox version 2.0.11 (www.statisticalmodelling.de/lst.html) for SPM. Lesion masks were estimated and then transformed into standard space and averaged to yield a mean lesion mask across subjects.

#### Brain Volume Analysis

Normalized brain volumes were quantified on high-resolution T1-weighted image using the SIENAx method, part of the FMRIB Software Library.^31^

### Statistical Analysis

The data were conducted using the SPSS Version 21.0 (SPSS, IBM Corporation, Chicago, IL). All tests were 2-sided, and significance was accepted at 5% level (alpha = 0.05). Regional cortical thickness analyses were exploratory in nature and thus we consider that the application of Bonferroni correction for multiple comparisons analysis (*P* < 0.05/33 number of comparisons in each hemisphere) would overcorrect for type I error, especially in view of the insufficient power derived from the small simple size. Therefore, the present study presents those regional cortical thickness results with a significance level *P* < 0.05 uncorrected. Nevertheless, since the probability of false-positive results is higher than the standard alpha value, the conclusions are based exclusively on criteria such as the magnitude and the impact of the differences (eg, number of patients who are at least 2 standard deviations [SDs] below the mean) rather than the *P* value.

Using the Shapiro-Wilk test, we determined that all variables except T2 white matter lesion volume were normally distributed (Shapiro-Wilk test, *P* > 0.05). To analyze the correlation among radiological measures (brain volumes, cortical thickness), and clinical parameters or T2 white matter lesion volume (not normally distributed), Pearson and Spearman coefficients were used respectively.

A general linear model anaysis of variance (ANOVA) was applied to determine statistically significant differences between the groups for the continuous variables. Age, sex, and educational level were included as covariates as they can contribute to cortical thickness.^32^ Homogeneity of variances assumption was not met (Levene test <0.05) for deep gray mater comparisons and therefore Welch F test was the approach for performing the ANOVA analysis, followed by post-hoc Games Howell test for mean pairwise comparison analyses. For the categorical variables, the k-independent samples Kruskall-Wallis test was applied with post-hoc analysis for Mann-Whitney *U* tests.

Differences in fractional anisotropy or mean diffusivity between groups were obtained by performing a voxel-based analysis by means of the general linear model, with age and sex as covariates. We tested the significance of any observed differences using a paired *t* test with *P* < 0.001.

## RESULTS

### Sample Characteristics

Table 1 summarizes the demographic and clinical characteristics of the entire simple and shows that the groups were well-matched for age (F[2,48] = 0.68, *P* = 0.51), sex ratio (*χ*^2^ = 0.55, *P* = 0.75), and educational level (*χ*^2^ = 0.08, *P* = 0.95). Reasons for the first RIS patients MRI, which was performed a mean of 4.07 years (range 1–11) earlier, were: headache (N = 5), dizziness (N = 4), tinnitus-hypocusia (N = 3), syncope (N = 1), restless legs (N = 1), research control (N = 1), traffic accident (N = 1), and prolactinoma (N = 1).

**TABLE 1:**
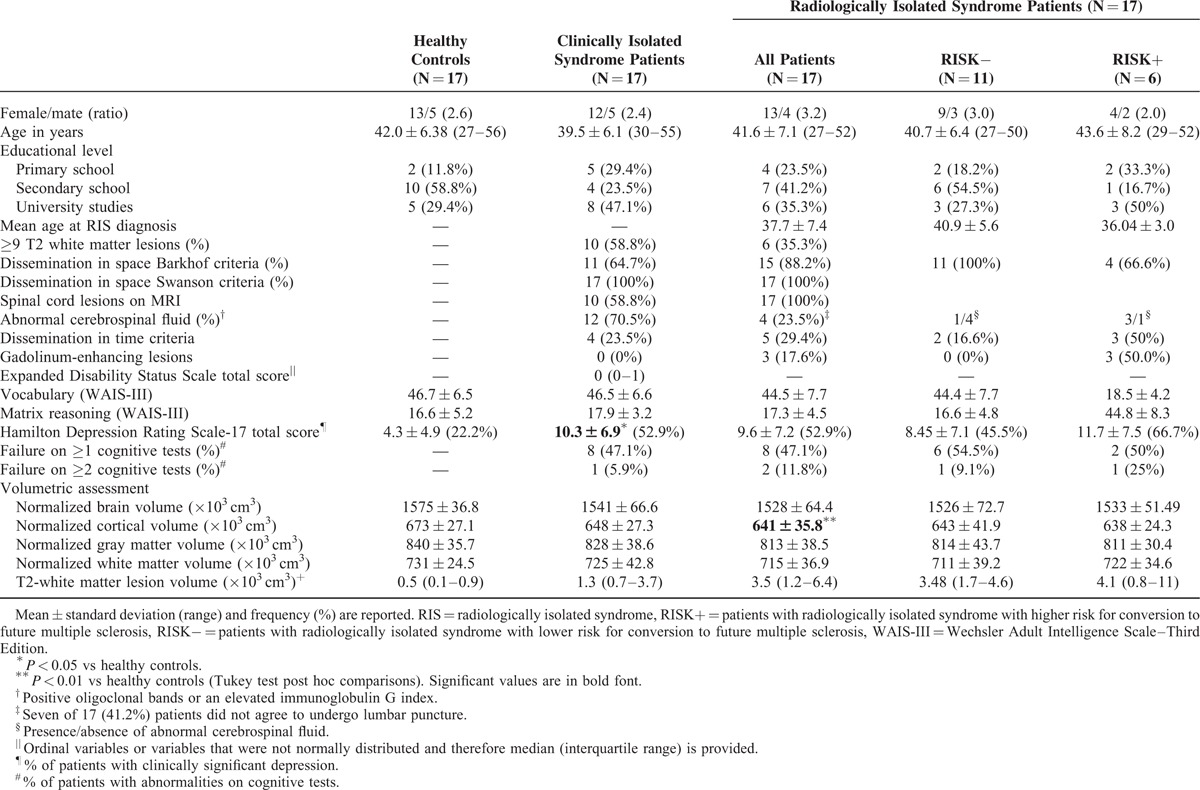
Demographic and Clinical Characteristics of the Sample

The RIS patients were stratified into RISK+ (higher risk for conversion to future MS) versus RISK− (lower risk for conversion to future MS) according to the presence of lesions within the spinal cord or no lesions of the spinal cord, but presence of at least 2 of the following characteristics: abnormal cerebrospinal fluid, gadolinium enhancing lesions, or dissemination in time. Among the 6 RIS subjects who were classified as RISK+, 4 were included based on spinal cord lesions criteria and 2 on the presence of several risk criteria for conversion to MS. No RIS patients were being treated with disease-modifying therapies.

CIS patients were characterized by low clinical disability (median EDSS score of 0, range 0–4) and a relatively short duration of disease (median disease duration from clinical onset = 12 months). Only 3 of 17 (17.6%) CIS patients had a disease evolution from clinical onset <12 months, whereas 7 of 17 (41.1%) had a disease duration from symptom onset of at least 48 months. Of the 17 CIS patients, 5 (29.4%) presented with spinal cord symptoms, 4 (23.5%) with optic neuritis, 3 (17.6%) with brainstem symptoms, 3 with polysimptomatic onset, and 2 (11.8%) with hemispheric cerebral symptoms. All CIS subjects fulfilled dissemination in space according to the McDonald 2010 criteria.^16^ Of the 17 CIS patients, only 4 (23.5%) were treated with disease-modifying therapies (interferon beta-1a).

Eight (47.1%) RIS patients failed in at least one cognitive test, defined as a *Z* score ≤2.0 SD below the healthy controls mean of any of the cognitive tests, whereas 2 (11.8%) RIS patients failed in at least 2 cognitive tests, proportions virtually identical to those observed in CIS patients (47.1% and 5.9% respectively).

Using a cutoff score of 8 on the Hamilton Depression Rating Scale total score,^22^ 9 (52.9%) of the RIS group had at least mild clinical depression, with more than half of them (N = 5, 55.9%) having moderate depressive symptoms, rates identical to that observed among CIS patients.

### Whole-brain Volume Analysis

Normalized cortical volumes were statistically different between groups as determined by ANOVA (F[2,48] = 5.22, *P* = 0.009). Normalized cortical volume was significantly lower on RIS group compared with healthy controls (673 ± 27.07 vs 641 ± 35.88 (cm^3^ × 10^3^), Tukey *P* test = 0.009), whereas near-significant difference was observed between CIS and healthy control groups (648 ± 27.34 vs 673 ± 27.07 (cm^3^ × 10^3^), Tukey *P* test = 0.057). A considerable trend toward significance in total brain volume was observed in RIS patients compared with healthy controls (1528 ± 64.40 vs 1575 ± 36.82, *P* = 0.054). Normalized total gray matter and white matter volumes were not statistically different between groups (F = 2.10, *P* = 0.13 and F = 0.94, *P* = 0.40, respectively). No differences between RISK− and RISK+ were detected. A trend toward lower normalized total brain and cortical volumes was observed in RIS patients who failed in at least one cognitive test compared with those without cognitive impairment, but these differences did not reach statistical significance (*P* = 0.12 and 0.33, respectively). No significant differences were found when RIS patients where subdivided according to the presence/absence of clinically significant depression (data not shown).

White matter lesion volume did not differ significantly between RIS and CIS groups as determined by Mann-Whitney *U* test (*P* = 0.13). T2-white matter lesion volume was correlated with normalized cortical volume (*r* = −0.59, *P* = 0.012) and normalized grey matter volume (*r* = −0.52, *P* = 0.03) in RIS patients. Among CIS patients, correlations were seen between T2-white matter lesion volume and normalized gray matter volume (*r* = −0.56, *P* = 0.020), normalized brain volume (*r* = −0.51, *P* = 0.038), and normalized cortical volume (*r* = −0.49, *P* = 0.045).

### Deep Gray Matter and Cerebellar Volumes

Between-groups comparisons on deep gray matter and cerebellum volumes are shown in Table 2. Significant group difference was found between groups with regard to thalamic volume (F Welch [2,30] = 4.61, *P* = 0.018). Post-hoc Games-Howell test indicated that this difference was because of a difference between healthy controls versus RIS (Games-Howell, *P* = 0.014). There were no other statistically significant differences between group means as determined by ANOVA Welch F test (*P* > 0.10 for all).

**TABLE 2:**
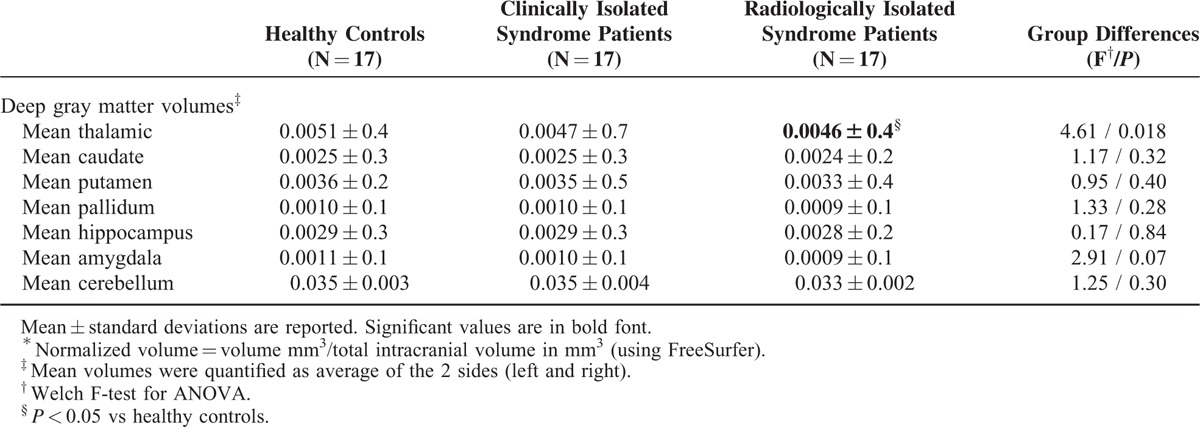
Normalized^∗^ Deep Gray Matter and Cerebellum Volumes (mm^3^) in Radiologically Isolated Syndrome and Clinically Isolated Syndrome Patients Compared With Healthy Controls

Among RIS patients, no correlations were found between mean thalamic volume and T2-white matter lesion volume (*r* = −0.31, *P* = 0.21), normalized brain (*r* = 0.42, *P* = 0.09) or normalized cortical volume (*r* = 0.45, *P* = 0.06).

### Cortical Thickness

Compared with healthy controls, RIS patients showed significant thinning in a number of cortical areas, that were primarily distributed in frontal and temporal lobes (*P* < 0.05, uncorrected). The extent of regional cortical thinning differed between the hemispheres, with most regions having the highest thinning in the right hemisphere. A single-subject analysis showed that right temporal lobe was particularly affected, as cortical thickness was at least 2 SDs below the healthy controls mean in the 47.1% of RIS subjects. Lateral orbitofrontal, transverse temporal gyrus, and posterior cingulate cortex were found significantly reduced bilaterally. After Bonferroni correction for multiple comparisons was applied (*P* < 0.0015), no significant thinning differences were found between groups in any regional cortical area.

Strong inverse correlations were observed between T2-white matter lesion volume and regional cortical thickness (rho spearman ranging from 0.5 to 0.78).

In comparison to the widespread cortical thickness differences between the RIS and healthy controls, those from the CIS and healthy controls were very limited (Table 3).

**TABLE 3:**
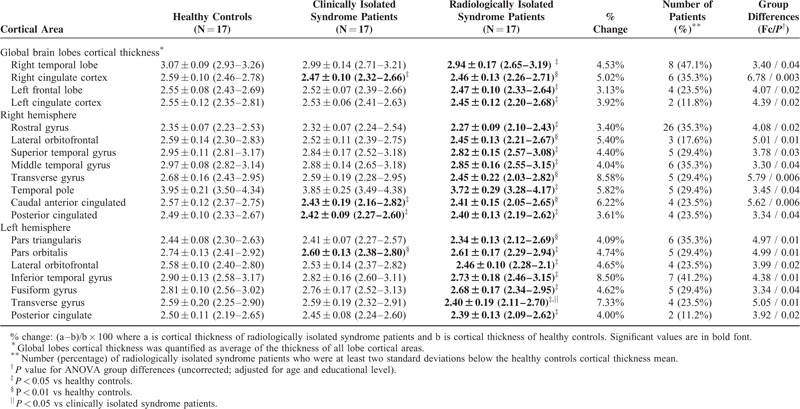
Cortical Areas Showing Significant Cortical Thinning in Radiologically Isolated Syndrome and Clinically Isolated Syndrome Patients Compared With Healthy Controls

### DTI

**FIGURE 1:**
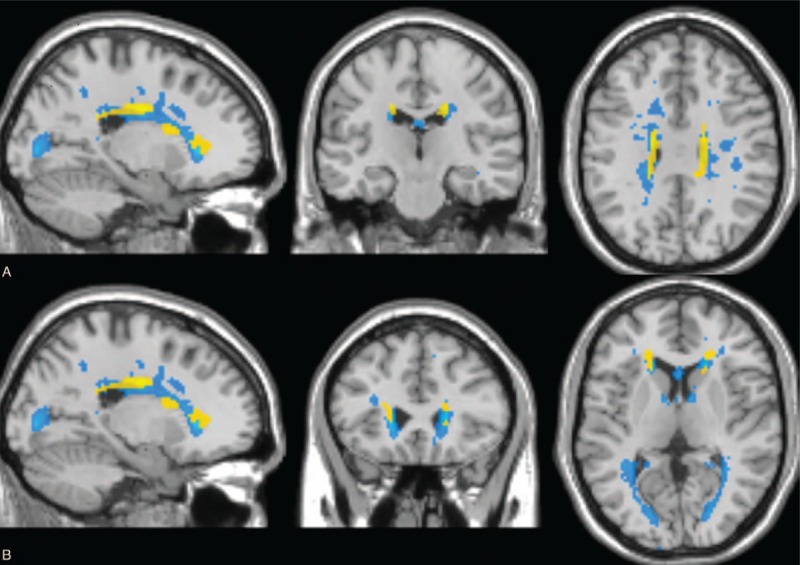
Voxel-based analysis results obtained using fractional anisotropy maps and mean lesion mask in radiologically isolated syndrome (RIS) patients. Saggital, coronal, and axial views are presented. Clusters of reduced fractional anisotropy in RIS patients compared with healthy controls (*P* < 0.001) are shown in yellow and average lesion mask is shown in blue. The overlay of the significant map clusters on the mean lesion mask shows that most of the abnormalities highlighted by voxel-based analysis were primary located within lesions. (A) Both cynculate gyri. (B) Bilateral frontal sub-gyral regions.

Voxel-based analysis results obtained using fractional anisotropy maps are illustrated in figure. Note that the clusters with altered microstructural integrity overlap with lesion mask are dominant in both cynculate gyri (Figure 1A) and in bilateral frontal sub-gyral regions of RIS patients (Figure 1B) (*P* < 0.001). A significant fractional anisotropy decrease in cerebellum white matter was found among CIS patients (*P* < 0.001). No significant fractional anisotropy or mean diffusivity changes was found in the normal-appearing gray matter of both RIS and CIS patients when compared with healthy controls. Detailed results, including T-maps values, number of voxels in each cluster, Montreal Neurological Institute coordinates, and *P* values are presented in Table 4.

**TABLE 4:**
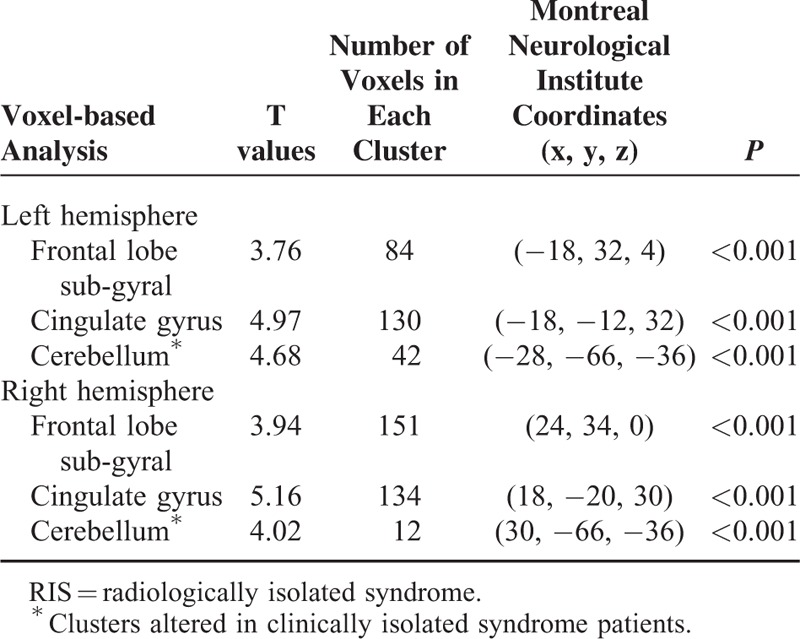
Voxel-based Morphometry Analysis of Reduced Fractional Anisotrophy in RIS Subjects Compared With Control Group

## DISCUSSION

Damage to both the normal-appearing white matter and gray matter has increasingly been recognized as a crucial component of MS.^33–35^ Motivated by the scant information regarding gray matter pathology in RIS patients, we investigated whether gray matter involvement occurs in RIS patients and whether it shares anatomical patterns with CIS patients by using multimodal 3T MRI approach.

In line with the findings of a recent RIS study performed using the same method of analysis,^10^ we found significant mean thalamic volume loss in RIS patients compared with healthy controls. This is consistent with previous research that showed that thalamic volume loss is one of the earliest and most prominent signs of gray matter pathology in MS.^36–38^ This finding might have a great clinical significance, as thalamic atrophy has been found as an independent predictor of the conversion to definite MS in patients with CIS.^39,40^ In the present study, thalamic atrophy was unrelated to the presence of other markers of evolution to MS. Future longitudinal studies are warranted to address whether thalamic atrophy in RIS patients may herald reliably the conversion to future MS. Unexpectedly, thalamic volume loss was more pronounced in RIS patients than in CIS patients. Thalamic atrophy, which has been shown to strongly correlated to white matter lesion volume,^41,42^ has been found primarily in those CIS patients who developed definite MS after a follow-up of 1 year.^38^ In contrast, CIS patients from our cohort, many of whom have a disease duration >12 months, have not yet developed definite MS and are characterized by median low white matter lesion volume, which certainly might contribute to the explanation of the current results.

RIS patients also exhibited significantly reduced total normalized cortical volume and regional cortical atrophy compared with healthy controls. Furthermore, the most striking finding of the present study was that both global cortical volume and regional cortical thickness were more pronounced in RIS patients than in CIS patients. Although these results were somewhat unexpected, they are indeed in line with previous research. Thus, although a large number of studies did not find decreased whole brain and cortical volumes in CIS patients,^36,37,39,43–45^ all the studies published so far (with the exception of one publication)^10^ have reported total brain and cortical atrophy in RIS patients,^3,6,8,9,12^ thereby suggesting that, in contrast to CIS patients, decreased cortical volume in RIS patients is highly consistent. However, there are also several reasons that might partially explain these results: CIS patients had a lower median white matter lesion volume compared with RIS (albeit not significant); although there was no significant age difference among CIS and RIS groups in the present study, RIS patients were approximately 2 years older than those from the CIS group; nearly one-third of the CIS patients presented with spinal cord symptoms, which might led to lower average cortical thickness (the type of clinical picture at onset has been found to correlate with atrophy in the corresponding cortical areas)^46^; in contrast with MS and CIS patients, the absence of a definable clinical event makes it impossible to establish the disease evolution among RIS patients and, thus, there is the possibility that RIS evolution have actually a much longer duration than expected.

Although marginal and no significant cortical thinning was observed among CIS patients, RIS patients showed significant thinning in a number of cortical areas that were primarily distributed in frontal and temporal lobes, as well as cingulate cortex. Although the differences did not reach statistical significance after Bonferroni correction, their magnitudes were meaningful, as evidenced by the fact that most cortical areas were at least 2 SD below the healthy controls cortical thickness mean in one-third of the RIS patients. Furthermore, average right temporal lobe cortical thickness was significantly reduced in nearly half of the RIS patients. Although no differences were found among RIS patients with presence/absence of other markers of evolution to MS (eg, spinal cord lesions), the small simple size did not allow us to draw conclusions with regard to this issue.

Interestingly, we observed reduced cortical thickness in particular areas of great clinical relevance. First, regional significant atrophy were present in anterior cingulate cortex and orbitofrontal cortex, regions that have an established role in the pathophysiology of emotion processing and regulation.^47^ Based on previous studies regarding clinical depression and its neurobiological basis,^48^ we hypothesized that these findings could represent a structural brain trait factor for developing psychological disorders, thereby providing a plausible biological explanation for the high rates of psychiatric disturbances observed in RIS patients.^4^ Second, posterior cingulate cortex was found to be significantly thinner bilaterally. Posterior cingulate cortex is known to be commonly affected by neurodegenerative diseases and has recently been showed to be particularly thinner in long-standing MS patients.^49^ In contrast, we did not observe cortical thickness in those brain regions that have been shown to play a critical role in motor function and physical dysfunction (precentral gyrus, sensorimotor cortex, and cerebellum), which contribute to explain why RIS patients subserve motor functions. Overall, these findings are totally in keeping with the clinical picture observed in RIS patients, characterized by null physical disability but high rates of cognitive and psychiatric disorders.^4,5^

To our knowledge, only one study has investigated cortical thickness in RIS patients.^10^ In that study,^10^ a limited area of thinning in the right superior and inferior parietal gyri was observed by using vertex-wise map corrected for multiple comparisons. The possible explanations for these discrepant results are mainly methodological issues, such as the patients’ characteristics at the study enrollment (no details about the presence of markers of evolution to MS or T2-white matter lesion volume were provided in the study of Azevedo),^10^ and the different statistical methods used to explore cortical thickness. In addition, 2 major problems inevitably related to RIS research might also contribute to find inconsistent results: the small sample sizes (an average of twenty patients) and the possibility that subjects fulfilling the RIS criteria actually constitute a highly heterogeneous group with different disease duration.

Another important finding of the present study is the observation that the voxel-based analysis did not reveal diffuse white matter fractional anisotropy or mean diffusivity changes in RIS patients. The results of the present study are in line with previous studies assessing microstructural integrity of white matter tracts in RIS patients by using magnetization transfer ratio^6^ and tract-based spatial statistics,^12^ further suggesting that occult microstructural normal-appearing white-matter damage is not significant present beyond T2 visible lesions in RIS patients. These results suggest that RIS patients might have experienced more efficient reparative mechanisms after white matter injury, thereby contributing to explain the lack of episodes of neurologic disturbance existing in RIS patients.

Very recent studies have demonstrated that white matter microstructural integrity is the strongest predictor for regional cortical atrophy in MS patients.^42,49,50^ Therefore, although the lack of diffuse normal-appearing white matter damage might additionally contribute to explain the absence of global and regional cortical atrophy observed in CIS patients, it raises the question whether cortical atrophy in RIS patients develops according to a distinct pathological process than in MS disease. Indeed, regional cortical thinning in RIS patients showed stronger inverse correlation to white matter lesion volume than that reported in MS research. A new study specifically designed to assess the association between RIS regional gray matter atrophy and both focal and diffuse pathology in anatomically connected white matter tracts is in progress, which will be helpful to properly address this issue.

The study should be interpreted within the context of several limitations. First, the sample size was relatively small. Given the low incidence and prevalence of the disease, the RIS literature generally comprises studies with small sample sizes.^6–10,12^ Second, we lacked for a method to visualize cortical lesions, the prevalence of which has been found to be as high as 40% among RIS patients,^7^ and, as a consequence, we were not able to assess their contribution to cortical thinning. However, cortical lesions in RIS patients have been shown not to be related to more pronounced cortical atrophy,^7^ which agrees with the conclusions of emerging research that suggest that cortical atrophy is largely independent of cortical demyelination.^51–53^ Third, MS lesions were not in-painted before submitting the image to FreeSurfer, which could compromise the accuracy of measured cortical thickness. However, according to a recent study,^54^ lesion in-painting has only a small effect on the estimated regional and global cortical thickness. Fourth, The CIS group enrolled in the present study was not homogeneous in terms of disease duration from initial demyelinating event. Fifth, cerebrospinal fluid data were only available in a small proportion of RIS patients. Sixth, the regional cortical thickness results were not corrected for multiple comparisons, which lead to an increase in probability of observing significant comparison just due to chance. However, the magnitude of the differences is meaningful (one-third of RIS patients are at least 2 SDs below the healthy controls mean in most of the significant thinning regional areas). Moreover, such results were not observed in CIS patients, further strengthening our findings.

Being conscious of the limits of our study, RIS patients here described are currently enrolled in a longitudinal clinical and MRI study, which will be helpful in demonstrating more confident results. Notwithstanding, our results suggest the existence of neuronal degeneration in RIS patients regardless of the presence of an initial demyelinating clinical episode, highlighting that RIS brain tissue damage might not be limited to focal white matter lesions. Additional studies with larger sample sizes are warranted for a better understanding of the pathophysiological processes underlying this novel entity.

## References

[1] Okuda DT Mowry EM Beheshtian A Incidental MRI anomalies suggestive of multiple sclerosis: the radiologically isolated syndrome. *Neurology* 2009; 72:800–805. 19073949 10.1212/01.wnl.0000335764.14513.1a

[2] Lebrun C Blanc F Brassat D Cfsep. Cognitive function in radiologically isolated syndrome. *Multiple sclerosis* 2010; 16:919–925. 20610492 10.1177/1352458510375707

[3] Amato MP Hakiki B Goretti B Association of MRI metrics and cognitive impairment in radiologically isolated syndromes. *Neurology* 2012; 78:309–314. 22262744 10.1212/WNL.0b013e31824528c9

[4] Labiano-Fontcuberta A Aladro Y Martinez-Gines ML Psychiatric disturbances in radiologically isolated syndrome. *J Psychiatr Res* 2015; 68:309–315. 26028549 10.1016/j.jpsychires.2015.05.008

[5] Labiano-Fontcuberta A Martinez-Gines ML Aladro Y A comparison study of cognitive deficits in radiologically and clinically isolated syndromes. *Multiple sclerosis* 2015. 10.1177/1352458515591072 26084350

[6] De Stefano N Stromillo ML Rossi F Improving the characterization of radiologically isolated syndrome suggestive of multiple sclerosis. *PloS one* 2011; 6:e19452. 21559385 10.1371/journal.pone.0019452 PMC3084867

[7] Giorgio A Stromillo ML Rossi F Cortical lesions in radiologically isolated syndrome. *Neurology* 2011; 77:1896–1899. 22076541 10.1212/WNL.0b013e318238ee9b

[8] Stromillo ML Giorgio A Rossi F Brain metabolic changes suggestive of axonal damage in radiologically isolated syndrome. *Neurology* 2013; 80:2090–2094. 23635962 10.1212/WNL.0b013e318295d707

[9] Rojas JI Patrucco L Miguez J Brain atrophy in radiologically isolated syndromes. *Journal of neuroimaging: official journal of the American Society of Neuroimaging* 2015; 25:68–71. 25307993 10.1111/jon.12182

[10] Azevedo CJ Overton E Khadka S Early CNS neurodegeneration in radiologically isolated syndrome. *Neurol Neuroimmunol Neuroinflamm* 2015; 2:e102. 25884012 10.1212/NXI.0000000000000102 PMC4396526

[11] Okuda DT Siva A Kantarci O Radiologically isolated syndrome: 5-year risk for an initial clinical event. *PloS one* 2014; 9:e90509. 24598783 10.1371/journal.pone.0090509 PMC3943959

[12] Giorgio A Stromillo ML De Leucio A Appraisal of brain connectivity in radiologically isolated syndrome by modeling imaging measures. *J Neurosci* 2015; 35:550–558. 25589750 10.1523/JNEUROSCI.2557-14.2015 PMC6605365

[13] Barkhof F Filippi M Miller DH Comparison of MRI criteria at first presentation to predict conversion to clinically definite multiple sclerosis. *Brain: a journal of neurology* 1997; 120 (Pt 11):2059–2069. 9397021 10.1093/brain/120.11.2059

[14] Tintore M Rovira A Martinez MJ Isolated demyelinating syndromes: comparison of different MR imaging criteria to predict conversion to clinically definite multiple sclerosis. *AJNR American journal of neuroradiology* 2000; 21:702–706. 10782781 PMC7976636

[15] Kurtzke JF Rating neurologic impairment in multiple sclerosis: an expanded disability status scale (EDSS). *Neurology* 1983; 33:1444–1452. 6685237 10.1212/wnl.33.11.1444

[16] Gomez-Moreno M Diaz-Sanchez M Ramos-Gonzalez A Application of the 2010 McDonald criteria for the diagnosis of multiple sclerosis in a Spanish cohort of patients with clinically isolated syndromes. *Multiple sclerosis* 2012; 18:39–44. 21865413 10.1177/1352458511417828

[17] Swanton JK Fernando K Dalton CM Modification of MRI criteria for multiple sclerosis in patients with clinically isolated syndromes. *J Neurol Neurosurg Psychiatry* 2006; 77:830–833. 16043456 10.1136/jnnp.2005.073247 PMC2117493

[18] Rao SM Leo GJ Bernardin L Cognitive dysfunction in multiple sclerosis. I. Frequency, patterns, and prediction. *Neurology* 1991; 41:685–691. 2027484 10.1212/wnl.41.5.685

[19] Bondi MW Serody AB Chan AS Cognitive and neuropathologic correlates of Stroop Color-Word Test performance in Alzheimer's disease. *Neuropsychology* 2002; 16:335–343. 12146681 10.1037//0894-4105.16.3.335

[20] Kaufman AS Lichtenberger EO The essentials of WAIS-III assessment. New York; Chichester: J. Wiley; 1999.

[21] de Oliveira MO Nitrini R Yassuda MS Vocabulary is an appropriate measure of premorbid intelligence in a sample with heterogeneous educational level in Brazil. *Behav Neurol* 2014; 2014:875960. 24803737 10.1155/2014/875960 PMC4006600

[22] Hamilton M A rating scale for depression. *J Neurol Neurosurg Psychiatry* 1960; 23:56–62. 14399272 10.1136/jnnp.23.1.56 PMC495331

[23] Talairach J Tournoux P Co-planar stereotaxic atlas of the human brain; 3-dimensional proportional system: an approach to cerebral imaging. New York, N.Y: Thieme Medical Publishers; 1988.

[24] Fischl B Salat DH Busa E Whole brain segmentation: automated labeling of neuroanatomical structures in the human brain. *Neuron* 2002; 33:341–355. 11832223 10.1016/s0896-6273(02)00569-x

[25] Fischl B Liu A Dale AM Automated manifold surgery: constructing geometrically accurate and topologically correct models of the human cerebral cortex. *IEEE Trans Med Imaging* 2001; 20:70–80. 11293693 10.1109/42.906426

[26] Desikan RS Segonne F Fischl B An automated labeling system for subdividing the human cerebral cortex on MRI scans into gyral based regions of interest. *NeuroImage* 2006; 31:968–980. 16530430 10.1016/j.neuroimage.2006.01.021

[27] Smith SM Fast robust automated brain extraction. *Hum Brain Mapp* 2002; 17:143–155. 12391568 10.1002/hbm.10062 PMC6871816

[28] Maes F Collignon A Vandermeulen D Multimodality image registration by maximization of mutual information. *IEEE Trans Med Imaging* 1997; 16:187–198. 9101328 10.1109/42.563664

[29] Ashburner J A fast diffeomorphic image registration algorithm. *NeuroImage* 2007; 38:95–113. 17761438 10.1016/j.neuroimage.2007.07.007

[30] Schmidt P Gaser C Arsic M An automated tool for detection of FLAIR-hyperintense white-matter lesions in Multiple Sclerosis. *NeuroImage* 2012; 59:3774–3783. 22119648 10.1016/j.neuroimage.2011.11.032

[31] Smith SM Zhang Y Jenkinson M Accurate, robust, and automated longitudinal and cross-sectional brain change analysis. *NeuroImage* 2002; 17:479–489. 12482100 10.1006/nimg.2002.1040

[32] van Velsen EF Vernooij MW Vrooman HA Brain cortical thickness in the general elderly population: the Rotterdam Scan Study. *Neurosci Lett* 2013; 550:189–194. 23831346 10.1016/j.neulet.2013.06.063

[33] Preziosa P Rocca MA Mesaros S Intrinsic damage to the major white matter tracts in patients with different clinical phenotypes of multiple sclerosis: a voxelwise diffusion-tensor MR study. *Radiology* 2011; 260:541–550. 21673227 10.1148/radiol.11110315

[34] Rovaris M Riccitelli G Judica E Cognitive impairment and structural brain damage in benign multiple sclerosis. *Neurology* 2008; 71:1521–1526. 18815387 10.1212/01.wnl.0000319694.14251.95

[35] Honce JM Gray matter pathology in MS: neuroimaging and clinical correlations. *Mults Scler Int* 2013; 2013:627870. 10.1155/2013/627870 PMC3708448 23878736

[36] Audoin B Davies GR Finisku L Localization of grey matter atrophy in early RRMS: A longitudinal study. *J Neurol* 2006; 253:1495–1501. 17093899 10.1007/s00415-006-0264-2

[37] Henry RG Shieh M Okuda DT Regional grey matter atrophy in clinically isolated syndromes at presentation. *J Neurol Neurosurg Psychiatry* 2008; 79:1236–1244. 18469033 10.1136/jnnp.2007.134825 PMC4827711

[38] Rocca MA Preziosa P Mesaros S Clinically isolated syndrome suggestive of multiple sclerosis: dynamic patterns of gray and white matter changes: a 2-year MR imaging study. *Radiology* 2016; 278:841–853. 26348234 10.1148/radiol.2015150532

[39] Calabrese M Rinaldi F Mattisi I The predictive value of gray matter atrophy in clinically isolated syndromes. *Neurology* 2011; 77:257–263. 21613600 10.1212/WNL.0b013e318220abd4

[40] Zivadinov R Havrdova E Bergsland N Thalamic atrophy is associated with development of clinically definite multiple sclerosis. *Radiology* 2013; 268:831–841. 23613615 10.1148/radiol.13122424

[41] Henry RG Shieh M Amirbekian B Connecting white matter injury and thalamic atrophy in clinically isolated syndromes. *J Neurol Sci* 2009; 282:61–66. 19394969 10.1016/j.jns.2009.02.379

[42] Steenwijk MD Daams M Pouwels PJ Unraveling the relationship between regional gray matter atrophy and pathology in connected white matter tracts in long-standing multiple sclerosis. *Hum Brain Mapp* 2015; 36:1796–1807. 25627545 10.1002/hbm.22738 PMC6869234

[43] Ceccarelli A Rocca MA Pagani E A voxel-based morphometry study of grey matter loss in MS patients with different clinical phenotypes. *NeuroImage* 2008; 42:315–322. 18501636 10.1016/j.neuroimage.2008.04.173

[44] Raz E Cercignani M Sbardella E Clinically isolated syndrome suggestive of multiple sclerosis: voxelwise regional investigation of white and gray matter. *Radiology* 2010; 254:227–234. 20019140 10.1148/radiol.2541090817

[45] Cappellani R Bergsland N Weinstock-Guttman B Diffusion tensor MRI alterations of subcortical deep gray matter in clinically isolated syndrome. *J Neurol Sci* 2014; 338:128–134. 24423584 10.1016/j.jns.2013.12.031

[46] Calabrese M Atzori M Bernardi V Cortical atrophy is relevant in multiple sclerosis at clinical onset. *J Neurol* 2007; 254:1212–1220. 17361339 10.1007/s00415-006-0503-6

[47] Drevets WC Price JL Furey ML Brain structural and functional abnormalities in mood disorders: implications for neurocircuitry models of depression. *Brain Struct Funct* 2008; 213:93–118. 18704495 10.1007/s00429-008-0189-x PMC2522333

[48] Bora E Fornito A Pantelis C Gray matter abnormalities in Major Depressive Disorder: a meta-analysis of voxel based morphometry studies. *J Affect Disord* 2012; 138:9–18. 21511342 10.1016/j.jad.2011.03.049

[49] Steenwijk MD Geurts JJ Daams M Cortical atrophy patterns in multiple sclerosis are non-random and clinically relevant. *Brain* 2016; 139 (Pt 1):115–126. 26637488 10.1093/brain/awv337

[50] Bergsland N Lagana MM Tavazzi E Corticospinal tract integrity is related to primary motor cortex thinning in relapsing-remitting multiple sclerosis. *Mult Scler* 2015; 21:1771–1780. 25791368 10.1177/1352458515576985

[51] van de Pavert SH Muhlert N Sethi V DIR-visible grey matter lesions and atrophy in multiple sclerosis: partners in crime? *J Neurol Neurosurg Psychiatry* 2015; Apr 29. pii: jnnp-2014-310142. doi: 10.1136/jnnp-2014-310142. [Epub ahead of print]. 10.1136/jnnp-2014-310142 PMC4853554 25926483

[52] Klaver R Popescu V Voorn P Neuronal and axonal loss in normal-appearing gray matter and subpial lesions in multiple sclerosis. *J Neuropathol Exp Neurol* 2015; 74:453–458. 25853695 10.1097/NEN.0000000000000189

[53] Popescu V Klaver R Voorn P What drives MRI-measured cortical atrophy in multiple sclerosis? *Mult Scler* 2015; 21:1280–1290. 25583833 10.1177/1352458514562440

[54] Govindarajan KA Datta S Hasan KM Effect of in-painting on cortical thickness measurements in multiple sclerosis: A large cohort study. *Hum Brain Mapp* 2015; 36:3749–3760. 26096844 10.1002/hbm.22875 PMC4839289

